# Investigation of dysmenorrhea in adolescent girls with familial Mediterranean fever: a comparative study with healthy controls

**DOI:** 10.1007/s00431-025-06033-8

**Published:** 2025-02-19

**Authors:** Fatma Gül Demirkan, Aylin Yetim Şahin, Figen Çakmak, Özlem Akgün, Vafa Guliyeva, Melike Zeynep Tuğrul Aksakal, Firdevs Baş, Nuray Aktay Ayaz

**Affiliations:** 1 Department of Pediatric Rheumatology, İstanbul School of Medicine, İstanbul University, İstanbul, Türkiye; 2 Department of Adolescent Medicine, İstanbul School of Medicine, İstanbul University, İstanbul, Türkiye

**Keywords:** Adolescents, Dysmenorrhea, Familial Mediterranean fever, Menstruation

## Abstract

Perimenstrual attacks have been reported in up to 15% of patients with FMF, suggesting that menstruation may be a trigger for FMF attacks. The aim of this study was to investigate menstrual period patterns and dysmenorrhea in adolescents with FMF in comparison to their healthy peers. This cross-sectional case–control study included 73 FMF patients and 70 age- and body mass index-matched controls. A structured questionnaire was designed to assess menstrual history, the frequency and severity of dysmenorrhea, symptoms related to dysmenorrhea, and the clinical features of FMF attacks. Dysmenorrhea was present in 90.4% of patients and 95.7% of controls (*p* = 0.32). Pain was reported during every cycle or every two cycles by 83.3% of patients versus 65.6% of controls (*p* = 0.02). Fever (27.4% vs. 10.3%, *p* = 0.01) was significantly more frequent in patients, while musculoskeletal symptoms (46.6% vs. 66.2%, *p* = 0.02), fatigue (53.4% vs. 83.8%, *p* < 0.001), and sleep disturbances (19.2% vs. 50.7%, *p* < 0.001) were more common in controls. Notably, FMF patients reported heavier bleeding episodes with higher number of sanitary pads used during menstruation (*p* = 0.001). Menstruation-associated FMF attacks were reported by 37% of patients, with 14.8% experiencing them every cycle. Exon 10 variants were present in 86.3% of cases, with 23.3% being homozygous. The frequency and character of dysmenorrhea did not differ significantly according to genetic profiles. *Conclusions*: This study is the first to investigate menstrual patterns and dysmenorrhea symptoms in adolescent FMF patients compared to their healthy peers. Dysmenorrhea is prevalent in FMF patients with distinct menstrual characteristics, including more frequent fever and heavier bleeding.

**Table:** 

**What is Known:** *• Menstruation may trigger Familial Mediterranean Fever (FMF) attacks in a subset of patients, but the relationship between FMF and dysmenorrhea remains unclear*. *• Some studies suggest that inflammation associated with FMF could contribute to menstrual pain and abnormalities, but comprehensive data in adolescents are limited*.	
**What is New:** *• This study is the first to compare menstrual patterns and dysmenorrhea characteristics between adolescent FMF patients and healthy controls, highlighting distinct menstrual symptoms in FMF patients*. *• FMF patients experience more frequent febrile episodes and heavier menstrual bleeding compared to their healthy peers, but dysmenorrhea characteristics are not influenced by specific MEFV gene mutations*.

**What is Known:**

*• Menstruation may trigger Familial Mediterranean Fever (FMF) attacks in a subset of patients, but the relationship between FMF and dysmenorrhea remains unclear*.

*• Some studies suggest that inflammation associated with FMF could contribute to menstrual pain and abnormalities, but comprehensive data in adolescents are limited*.

**What is New:**

*• This study is the first to compare menstrual patterns and dysmenorrhea characteristics between adolescent FMF patients and healthy controls, highlighting distinct menstrual symptoms in FMF patients*.

*• FMF patients experience more frequent febrile episodes and heavier menstrual bleeding compared to their healthy peers, but dysmenorrhea characteristics are not influenced by specific MEFV gene mutations*.

## Introduction

Familial Mediterranean fever (FMF) is an inherited autoinflammatory disease characterized by recurrent and self-limiting attacks that primarily affects individuals of Mediterranean descent [1]. Common features include fever, abdominal/chest pain, erysipelas-like erythema (ELE), and arthritis [2, 3].

The menstrual cycle, a complex physiological process governed by hormonal alterations, plays a crucial role in the reproductive health of adolescent girls and women [4, 5]. Many teenage girls (nearly 95%) experience menstrual pain (dysmenorrhea) during their periods [6, 7]. Over a third of them report severe pain, which often leads to missing school [8] or disrupting their ability to focus and learn [6].

It is stated that the etiology of dysmenorrhea is multifactorial. Genetic predisposition, body mass index (BMI), nutrition, physical activity, and psychosocial factors are some of the influencing factors [9]. In the literature, there are conflicting results especially in studies examining the relationship between BMI and dysmenorrhea [10]. It has been reported that dysmenorrhea may increase in adolescents with low BMI due to hormonal imbalances and insufficient fat tissue affecting estrogen production, while dysmenorrhea frequency may increase in those with high BMI due to increased prostaglandin levels by increased cytokine secretion [11–13].

The existing literature indicates that the hormonal milieu during menstruation may contribute to the development of inflammation, trigger attacks in individuals with FMF, and potentially influence disease severity [14–17]. Since 1960, studies have examined patients with FMF attacks that coincide with menstruation or are confined to the menstrual period, revealing that 7–15% of women with FMF experience such attacks [15, 18]. In addition, several recent studies have also shown that patients whose FMF attacks are associated with menstrual bleeding have higher rates of dysmenorrhea and frequent premenstrual and postmenstrual attacks than patients whose attacks are not associated with menstrual bleeding [19, 20]. The presence of fever and other systemic symptoms accompanying abdominal pain and failure of NSAID treatment in such patients are considered as important clues in the diagnosis of perimenstrual FMF attacks. However, research exploring the connection between menstruation-associated attacks and dysmenorrhea in FMF patients remains still limited.

The aim of this study was to describe the characteristics of menstrual cycles in adolescent girls with FMF and make comparisons with healthy peers. Furthermore, we sought to identify the unique characteristics of dysmenorrhea in adolescents with FMF and to examine how disease-related factors influence menstrual cycles.

## Materials and methods

### Participants

This cross-sectional observational study included adolescent girls with FMF who met the Eurofever/ Pediatric Rheumatology International Trials Organization (PRINTO) criteria based on clinical features and genetic testing (*n* = 73) and age and BMI matched healthy controls (*n* = 70) [1]. Healthy control group was randomly selected from adolescents who visited the adolescent health clinic for routine health evaluations and were not receiving follow-up care at any other clinic. Exclusion criteria were as follows: (1) presence of additional diseases and medication use other than colchicine or anti-interleukin (IL) 1 drugs for FMF, (2) presence of secondary dysmenorrhea, (3) history of previous pelvic or abdominal surgery, and (4) premenarcheal girls.

BMI of the participants was calculated as a ratio of body weight (kg) to height squared (m^2^) and SDS using national data. BMI data was categorized as follows: underweight (< − 2 SDS), normal weight (− 2 and + 1 SDS), overweight (+ 1 and + 2 SDS), and obese (> + 2 SDS) [21, 22].

Primary dysmenorrhea has been described as lower abdominal cramping pain that occurs just before or at the onset of menstruation and can last up to 3 days in the absence of underlying medical conditions [23, 24].

### Study design

A structured self-administered questionnaire was designed to gather detailed data on demographics and clinical features of menstrual periods of participants. The questionnaire included sections addressing the mother’s menarche age, clinical aspects of menstrual cycles (e.g., age at menarche, menorrhagia, metrorrhagia, number of pads used, and the regularity of the menstrual cycle), need of analgesics during periods, frequency, localization, and character of dysmenorrhea, symptoms accompanying dysmenorrhea, history of hospital admission or hospitalization due to dysmenorrhea, effect on quality of life, school/exam absenteeism due to dysmenorrhea. The patient group was also asked about their ability to differentiate FMF attacks from dysmenorrhea and the frequency and clinical characteristics of FMF attacks coinciding with menstrual periods. According to definitions in the literature, FMF-related attacks were identified in patients who did not respond to NSAIDs, had elevated AFR levels during the attack, or presented with other FMF symptoms such as fever [19, 25]. While preparing the survey questions, the literature on dysmenorrhea and related symptoms in the existing literature on the subject was taken into consideration [15, 19, 20, 25].

In the patient group, information on Mediterranean fever (*MEFV*) gene profile, attack characteristics, colchicine use, and use of anti IL-1 therapy in case of colchicine resistance were recorded. Colchicine resistance was characterized by experiencing one or more attacks each month for a duration of three months, or by the existence of subclinical inflammation despite receiving the highest tolerable dose of colchicine [26]. The variant analysis of the *MEFV* gene, performed by Sanger sequencing, was obtained from the files of the patients.

### Data collection

The survey was administered to patients diagnosed with FMF who were seen at the tertiary pediatric rheumatology clinic and to healthy teenage girls who were seen at the adolescent unit from February to August 2024. Responses were anonymized to maintain participant confidentiality.

### Statistical analysis

The sample size was established through G power analysis and informed by prior research. Considering the number of subjects in the reference studies to obtain a strong effect size (*d* = 0.5) for the groups, at least 128 subjects (at least 64 subjects for each group) were included in the study with a power of 80% and a confidence level of 95% [19].

The data were presented as numbers and percentages for categorical variables, while numeric variables were summarized using mean, median, and interquartile range (IQR). The distribution of the variables was evaluated using the Kolmogorov–Smirnov and Shapiro–Wilk tests. For the analysis of quantitative independent data, independent sample *t*-tests, Kruskal–Wallis tests, and Mann–Whitney *U* tests were employed. Categorical data were evaluated using the chi-square test. SPSS (Statistical Package for Social Sciences) version 26.0 was utilized for statistical analyses and visualized with GraphPad Prism, version 10.0 (GraphPad). All tests were two-tailed, with a significance level set at *p* < 0.05.

### Ethical considerations

The study adhered to the principles outlined in the Declaration of Helsinki. Informed consent was obtained from all participants or parents/guardians of the children. The research protocol was approved by the local institutional ethics committee of Istanbul University, Istanbul School of Medicine. (Date and number: 19.01.2024–2352229).

## Results

### Demographics, clinical characteristics, and genetic profile of the patients

The median age of patients at last visit was 15 (13–17) years, while the median age of onset and diagnosis was 6.3 (4–8) and 8 (6–10) years, respectively. A family history of FMF was present in 37% of the patient group. Among genetic profile, an exon 10 mutation variant was identified in 86.3% of patients, with 23.3% having a homozygous exon 10 mutation variant.

Regarding the symptoms during attacks, recurrent abdominal pain (65.8%) and fever (54.8%) were the most common two, while less frequent symptoms included diarrhea (4.1%) and pericarditis (1.4%). Ten (13.7%) patients reported at least one attack in the past 6 months.

Colchicine resistance was observed in 6.8% of cases, and amyloidosis or other serious complications were not reported. Additionally, no medication noncompliance was observed in any of the patients. Table 1 summarizes the demographic and clinical data and genetic profiles of the patients.

**Table 1:** Characteristics of familial Mediterranean fever patients

**Demographics**	
Median age of onset (year) (IQR 25–75)	6.3 (4–8)
Median age at diagnosis (year) (IQR 25–75)	8 (6–10)
Median duration of diagnostic delay (month) (IQR 25–75)	12 (3–24)
Median age at last visit (year) (IQR 25–75)	15 (13–17)
Median number of attacks (n) (IQR 25–75)	8 (4–12)
Presence of inbreeding (*n*, %)	10 (13.7)
Family history of FMF (*n*, %)	27 (37)
Family history of amyloidosis (*n*, %)	3 (4.1)
**Characteristics** **of** **attacks** (*n*, %)	
Fever	50 (68.5)
Abdominal pain	60 (82.2)
Constipation	5 (6.8)
Diarrhea	4 (5.5)
Chest pain	17 (23.3)
Pericarditis	2 (2.7)
Arthralgia	48 (65.8)
Arthritis	21 (28.8)
Exertional leg pain	13 (17.8)
Myalgia	16 (21.9)
Protracted febrile myalgia	2 (2.7)
ELE	11 (15.1)
Amyloidosis	0
Colchicine resistance (*n*, %)	7 (9.6)
MEFV gene profile (*n*, %)	
Exon10 (homozygous)	17 (23.3)
Exon10 (compound heterozygous)	12 (16.4)
Exon10 (heterozygous)	20 (27.4)
Exon10-Exon2 (compound heterozygous)	14 (19.2)
Exon2 (homozygous)	1 (1.4)
Exon2 (heterozygous)	9 (12.3)

*FMF*, familial Mediterranean fever; *IQR*, interquartile range; *ELE*, erysipelas-like erythema; *MEFV*, MEditerranean FeVer

### Characteristics of dysmenorrhea in adolescents with FMF

In the patient cohort, 66 (90.4%) patients had dysmenorrhea, and 7 (9.6%) patients had no signs of dysmenorrhea. No significant differences were found in age of onset, age at diagnosis, duration of diagnostic delay, or occurrence of FMF attacks between these two groups.

When the course of abdominal pain during dysmenorrhea was asked, 10 patients (13.7%) described constant, fixed pain, while 56 patients (74%) described cramp-like pain with fluctuations.

The frequency of dysmenorrhea did not differ significantly between patients with homozygous or heterozygous exon 10 variants and patients with variants outside exon 10. We performed a sub-analysis comparing classical homozygous FMF cases to controls and found no significant differences in the frequency or characteristics of dysmenorrhea between the groups. Colchicine-responsive and colchicine-resistant patients had similar rates of dysmenorrhea.

### Characteristics of menstrual cycle associated attacks

Approximately three-quarters of the patients (76.7%) reported that they could distinguish between the symptoms of an FMF attack and the menstrual cycle, and 27 (37%) of them reported experiencing FMF attacks simultaneously with their menstrual periods.

Of 27 patients who experienced attacks with menstrual cycles, 14.8% (*n* = 4) experienced an attack in every cycle, 22.2% (*n* = 6) in every two cycles, and 29.6% (*n* = 8) in every 3–4 cycles, and in the remaining cases (*n* = 9), simultaneous attacks and menstruation were extremely rare.

Homozygous exon 10 variants were present in 9 (33.3%) and heterozygous exon 10 variants were present in 7 (24.9%) of the patients who experienced FMF attacks related with menstrual bleeding. Patients with an exon 2 heterozygous variant (*n* = 4/27, 14.8%) or compound heterozygous with an exon 10 variant (*n* = 4/27, 14.8%), were less likely to report such episodes.

**Fig. 1:**
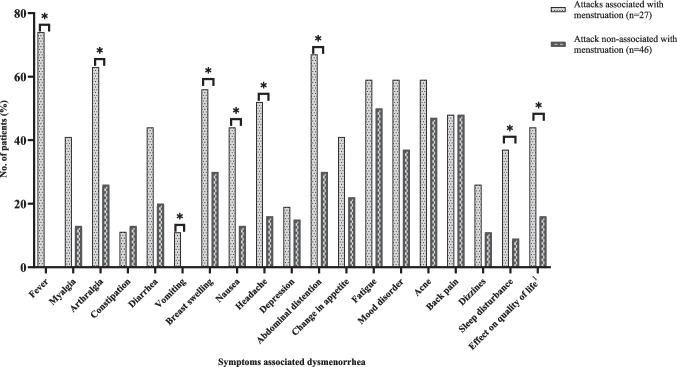
Distribution of symptoms accompanying dysmenorrhea in patients with and without menstruation associated FMF attacks who had dysmenorrhea during menstrual bleeding; *p < 0.05 statistically significant difference. ^1^Includes responses quite limited and limited for the effect of dysmenorrhea on quality of life

There was no difference in the incidence of dysmenorrhea in adolescents with (*n* = 26, 96.3%) and without (*n* = 40, 87.7%) menstruation-associated FMF attacks. Patients with menstruation- associated FMF attacks were more likely to have features of fever, arthralgia, abdominal distention, sleep disturbances, headache, nausea, vomiting, and breast swelling associated with dysmenorrhea than those who did not have menstruation- associated FMF attacks **(**Fig. 1**)**. Seven patients with menstruation- associated attacks reported no episodes of fever in association with dysmenorrhea. Among them, four experienced arthritis and arthralgia, and one reported chest pain without abdominal pain during episodes. The remaining two patients stated that they distinguished between FMF attacks and dysmenorrhea symptoms by noting the lack of response to analgesics and the localization of the pain.

### Comparison of menstrual period characteristics of the patient and control groups

There was no difference in age at menarche between patients and controls (*p* = 0.28). The groups were similar in terms of duration of menstrual bleeding and length of intermenstrual intervals. The number of pads used in the first 3 days of menstruation was significantly less in the control group (*p* = 0.001). The comparison of menstrual cycle characteristics is outlined in Table 2.

**Table 2:** Demographic data and menstrual characteristics of the groups

Characteristics	Control (*n* = 70)	Patients (*n* = 73)	*p*
Median age (year) (IQR 25*–*75)	15 (13***–***16)	15 (13***–***17)	0.78
Median BMI (kg/m^2^) (IQR 25*–*75)	21.11 (19.13***–***23.64)	20.7 (19.22***–***22.21)	0.19
Median age of menarche (year) (IQR 25*–*75)	12 (11***–***13)	12 (11***–***13)	0.28
Median duration of menstruation (day) (IQR 25*–*75)	5 (5***–***7)	6 (5***–***7)	0.15
Median duration between cycles (day) (IQR 25*–*75)	28 (28)	28 (24***–***30)	0.93
Irregularity of periods (*n*, %)	7 (10)	12 (16.4)	0.32
Pad number in the first three days of menstruation (*n*) (median, IQR 25*–*75)	2.5 (2***–***3.2)	8 (4***–***10.5)	0.001*

*BMI*, body mass index; *SD*, standard deviation; *IQR*, interquartile range

* and bold values indicate statistical significance at *p* < 0.05

The frequency of dysmenorrhea was over 90% in both patients (90.4%) and controls (95%). Frequency of dysmenorrhea and positive family history of dysmenorrhea were similar between groups (*p* = 0.32, *p* = 0.5, respectively).

When asked about the timing of pain, a significantly higher proportion of the control group reported more pain during menstruation (*p* = < 0.001), patients complained about more pain just before and both before and during menstruation (*p* = < 0.001). The comparison of the frequency of painful menstruation cycles showed a significant difference with 83.3% (*n* = 55) of the patients and 65.6% (*n* = 44) of the control group having pain in every period or every two periods (*p* = 0.02). Patients and controls exhibited similar patterns of pain localization during dysmenorrhea, with the back/lumbar region and/or lower abdomen being the most commonly reported areas. Responses to the question assessing symptoms associated with dysmenorrhea revealed that musculoskeletal symptoms, including arthralgia and myalgia (*p* = 0.02), headache (*p* = 0.07), fatigue (*p* < 0.001), and sleep disorders (*p* < 0.001), were significantly more prevalent in the control group. In contrast, fever (*p* = 0.01) was significantly more common in the patient group. In both groups, very few patients had history of hospital admission or required hospitalization due to dysmenorrhea.

When asked about the impact of dysmenorrhea on daily life, around one-third (*n* = 24, 35.8%) of the control group and one-quarter (*n* = 18, 27.3%) of the patient group reported feeling limited or quite limited on the *Likert scale*. No differences were found between the groups in terms of frequency of school and exam absences due to dysmenorrhea (Table 3**)**.

**Table 3:** Comparison of dysmenorrhea characteristics of the groups

	Control (*n* = 70)	Patients (*n* = 73)	*p*
Presence of family history for dysmenorrhea (*n*,%)	46 (66.7)	53 (72.6)	0.47
Presence of dysmenorrhea (*n*,%)	67 (95.7)	66 (90.4)	0.32
Time of dysmenorrhea (*n*,%)			< 0.001*
During menses	63 (94.1)	11 (16.6)	< 0.001*
Prior to menses	4 (5.9)	25 (37.9)	< 0.001*
Both prior to and during the menses	0 (0)	30 (45.5)	< 0.001*
Localization (*n*,%)			0.23
Low abdomen	56 (83.6)	46 (69.7)	
Back/lumbar	10 (14.9)	18 (27.3)	
Thigh	1 (1.5)	1 (1.5)	
Leg	0 (0)	1 (1.5)	
Frequency (*n*,%)			0.04*
Every period	38 (56.8)	37 (56.1)	0.5
Every two periods	6 (8.9)	18 (27.2)	0.02*
In at least 1*–*2 periods per year	23 (34.3)	11 (16.7)	0.04*
Absenteeism due to dysmenorrhea (*n*,%)			0.34
Very frequently-frequently	5 (7.4)	10 (15.1)	
Sometimes	10 (14.9)	13 (19.7)	
Rarely	19 (28.4)	13 (19.7)	
Never	33 (49.3)	30 (45.5)	
Absenteeism for quiz due to dysmenorrhea (*n*,%)			0.32
Usually	0 (0)	3 (3) (4.5)	
Sometimes	6 (9)	7 (10.6)	
Rarely	7 (10.4)	7 (10.6)	
Never	54 (80.6)	49 (74.3)	
Duration of dysmenorrhea (*n*,%)			0.71
< 1 day	17 (25.4)	23 (34.8)	
1*–*3 days	39 (58.2)	34 (51.5)	
3*–*5 days	8 (11.9)	7 (10.7)	
> 5 days	3 (4.5)	2 (3)	
Hospital admission for dysmenorrhea (*n*,%)	2 (2.9)	6 (8.2)	0.27
Hospitalization for dysmenorrhea (*n*,%)	2 (2.9)	1 (1.4)	0.61
Analgesic use (*n*,%)	31 (47)	34 (56.7)	0.29
Accompanying symptoms of dysmenorrhea (*n*,%)			
Fever	7 (10.3)	20 (27.4)	0.01*
Musculoskeletal symptoms	45 (66.2)	34 (46.6)	0.02*
Constipation	12 (17.6)	9 (12.3)	0.37
Diarrhea	14 (20.6)	21(28.8)	0.26
Vomiting	8 (11.8)	3 (4.1)	0.12
Breast swelling and tenderness	29 (42.6)	29 (39.7)	0.73
Nausea	29 (42.6)	18.(24.7)	0.32
Headache	40 (58.8)	26 (35.6)	0.007*
Abdominal distension	38 (55.9)	32 (43.8)	0.17
Fatigue	57 (83.8)	39 (53.4)	< 0.001*
Sleep disturbance	34 (50.7)	14 (19.2)	< 0.001*
Effect on daily life (*n*,%)			0.13
Quite limited	6 (8.9)	3 (4.5)	
Limited	18 (26.9)	15 (22.8)	
Rarely limited	35 (52.3)	31 (46.9)	
Never	8 (11.9)	17 (25.8)	

^*^ bold values indicate statistical significance at *p* < 0.05

## Discussion

This study undertook a comprehensive examination of the relationship between FMF attacks and menstrual cycle characteristics in adolescent girls, highlighting the occurrence and intensity of dysmenorrhea, its association with genetic variants, and its overall impact on quality of life. The findings revealed that dysmenorrhea was prevalent among both FMF patients and healthy controls. Patients had significantly heavier menstrual bleeding compared with controls, but the groups were similar in terms of duration of menstrual bleeding and length of intermenstrual periods. Moreover, the menstrual cycles with dysmenorrheal were more frequent in adolescent girls with FMF than the controls. These results contribute to the expanding literature on the relationship between FMF and dysmenorrhea, providing notable data particularly relevant to adolescent patients with FMF.

The cyclical nature of menstruation may complicate the clinical presentation of FMF, as hormonal fluctuations in estrogen and progesterone are thought to influence the inflammatory response and trigger FMF attacks during periods [15, 17, 19, 27, 28]. In 2001, Ben-Chetrit et al. reported that 10 out of 141 female FMF patients (7%), two of whom were adolescents, experienced attacks triggered by menstruation. They noted that this phenomenon was unrelated to the patient’s age, disease duration, or the specific variants causing FMF. The authors suggested that the significant decrease in estrogen levels during menstruation, together with its waning protective effects, may contribute to acute FMF attacks [15]. Our findings align with this hypothesis, as nearly one-third of FMF patients experienced menstruation-associated attacks. In 2013, Karadağ et al. conducted a study involving 275 FMF patients, including 98 females, to investigate factors triggering attacks with serositis or musculoskeletal pain. They found that 33.7% of female patients reported menstruation-associated FMF attacks [17]. Similarly, in the study by Kishida et al., which aimed to identify factors triggering febrile attacks in Japanese FMF patients, 91 out of 229 female patients (39.7%) experienced attacks associated with menstruation [5]. The study by Akar et al. not only focused on the relationship between menstrual cycles and FMF attacks but also assessed the course of pregnancy in FMF patients [25]. Thirty-eight patients (53%) reported that their attacks frequently coincided with their menstrual cycles and 17 patients noticed pleuritic chest pain in addition to their abdominal attacks. During the pregnancies, 25 patients (62.5%) experienced complete symptomatic remissions. Additionally, Batu et al. reported a recent study on 151 adolescent patients, revealing that 23.2% of patients experienced attacks associated with menstruation [19]. They also reported that on-demand therapies (such as starting colchicine, increasing the colchicine dose, switching from coated to compressed colchicine tablets, adding anti-IL 1 drugs, or using on-demand non-steroidal anti-inflammatory drugs, glucocorticoids, and anakinra) were effective in controlling menstruation-associated attacks.

It is considered that decreased estrogen levels are not the only culprit in triggering FMF attacks during menstruation. In addition to eliminating the protective effect of estrogen in menstruation cycles, the metabolism of colchicine and estrogen by the same liver enzyme, cytochrome P450 3A4, also plays a role in triggering attacks [29]. During menstruation, the decrease in estrogen levels may increase the enzyme’s availability for colchicine metabolism, potentially lowering colchicine concentrations and reducing its protective efficacy. This interplay between hormonal changes and colchicine metabolism offers a possible explanation for the link between menstruation and FMF attacks, though further studies are needed to confirm this hypothesis. Recognition of FMF attacks associated with the menstrual cycle highlights the need for integrated care approaches that address both inflammatory and hormonal factors. Recently, Magnotti et al. reported a chemical screen which identified sex hormone catabolites as pyrin activators [30]. The results suggested that FMF patients display a moderate increase in steroid catabolite-induced inflammasome responses that could contribute to inflammatory flares and be dependent on the *MEFV* genotype. Moreover, authors discussed that the increase in pregnanolone may lower pyrin inflammasome threshold toward the end of pregnancy and during menstruation. Despite the demonstrated role of progesterone in pyrin metabolism and the abovementioned effects of estrogen, these assertions do not explain why only a small number of women experience attacks during their menstrual period and suggest that many factors may play a role in this process.

Our study provides important contributions in that it was conducted in the adolescent age group and evaluated together with the control group. Furthermore, the disease character and the relationship between attacks and dysmenorrhea were also examined within the patient group. As a result, patients with menstruation- associated FMF attacks were more likely to exhibit non-classical symptoms of FMF flares, such as breast swelling and acne during dysmenorrhea than those who did not have menstruation- associated FMF attacks. The observed differences in non-classical symptoms of FMF further underscore the complex interplay of factors involved and such detailed evaluations have not been found in the existing literature. Future research should focus on identifying additional mechanisms and individual susceptibilities to better understand and manage FMF attacks during the menstrual cycle.

Women with FMF may experience more frequent and severe dysmenorrhea, potentially linked to the inflammatory processes triggered by *MEFV* variants [31–33]. Our study found no difference in dysmenorrhea frequency compared to healthy controls. However, painful menstrual cycles were significantly more common in patients (83.3%, *n* = 55) than in the control group (65.6%, *n* = 44) (*p* = 0.02). Additionally, FMF patients had a significantly higher rate of symptoms starting before menstruation, possibly indicating more severe dysmenorrhea. Although these findings did not establish a direct link with the MEFV gene, they indicate that the intensity and severity of dysmenorrhea may be higher in FMF patients compared to healthy adolescents. Several studies have examined the role of the *MEFV* gene in the association between FMF and dysmenorrhea and have focused on the disease as a factor that triggers dysmenorrhea among adults [20, 34]. In 2013, Erten et al. hypothesized that adults with dysmenorrhea could have a higher prevalence of *MEFV* gene variants [20]. They reported an increased frequency of *MEFV* variants in patients with primary dysmenorrhea and speculated that these patients may subsequently develop additional clinical manifestations of FMF. Similarly, a study conducted on 1000 adult women also provided evidence that FMF should be considered in the etiology of dysmenorrhea that does not respond to non-steroidal anti-inflammatory drugs [34]. The study included women presenting to the emergency department with recurrent abdominal pain, particularly during the menstrual period. The number of individuals with a variant in at least one *MEFV* allele was 511 (51.1%). Our study stands out with its distinct design, as it is the first to compare adolescent FMF patients and healthy controls regarding dysmenorrhea and related symptoms, without assessing *MEFV* variants in the control group. Additionally, one of the most important findings of this study is the absence of an association between exon 10 variant and dysmenorrhea features, regardless of whether the variant is homozygous or heterozygous. Therefore, our findings indicate that there is no definitive link between genetic variants and dysmenorrhea. Further research and increased knowledge in this area are required to address these uncertainties.

Beyond severe pain, heavy bleeding during menstruation can impact health and quality of life. In the present study, the significantly higher number of sanitary pads used during the first three days of menstruation in FMF patients suggests heavier menstrual bleeding compared to controls. This finding is consistent with studies showing that systemic inflammation may increase menstrual flow by affecting vascular permeability and uterine contractility [35, 36]. In 2022, Orlandi et al. evaluated gynecological symptoms and disorders in women with rheumatic diseases including connective tissue diseases, arthritis, and spondyloarthritis [32]. Compared to the control group, these women reported significantly higher rates of heavy menstrual bleeding during both adolescence and adulthood. The increased menstrual bleeding observed in FMF patients, together with similar findings in other inflammatory diseases, highlights the potential impact of chronic inflammation on menstrual health and the importance of considering gynecological symptoms in the management of these patients.

The prevalence of dysmenorrhea exceeded 90% in both FMF patients and controls, consistent with global data suggesting high rates of dysmenorrhea among adolescent females [7, 19, 20, 23, 24, 34]. However, our study found notable differences in the timing of dysmenorrhea. While dysmenorrhea in healthy controls predominantly occurred during menstruation, FMF patients experienced pain both before and during menstruation. These findings align with the inflammatory hypothesis, as FMF-related systemic inflammation may amplify menstrual pain throughout the cycle. This observation echoes findings from studies that reported heightened inflammatory activity during FMF attacks, which could overlap with the inflammatory processes of menstruation [31, 33].

The frequency of dysmenorrhea in the patient group, with many experiencing pain in nearly every cycle, contrasts with the control group, where the majority reported less frequent painful cycles. This observation could suggest that individuals with more frequent dysmenorrhea may have underlying heightened prostaglandin sensitivity, which has been documented in the literature as a contributing factor to more severe and frequent menstrual pain [24]. Prostaglandins, key mediators of inflammation, are likely more active in FMF patients due autoinflammatory nature of disease [37]. Although specific studies on prostaglandin sensitivity during the menstrual cycle in FMF patients are lacking, the dysregulated inflammasome and interleukin-1 overproduction in FMF likely amplify inflammatory responses, potentially enhancing prostaglandin activity.

Interestingly, while many symptoms linked to dysmenorrhea, such as fatigue, headaches, musculoskeletal symptoms, and sleep disturbances, were significantly more prevalent in the control group, fever and diarrhea were more common in the patient group. The lower frequency of most symptoms accompanying dysmenorrhea in the patient group may be attributed to FMF patients experiencing painful attacks like serositis, which alters their pain perception and raises their pain thresholds compared to healthy individuals. Moreover, the regular follow-up and symptom monitoring that patients receive in outpatient clinics likely enhance their awareness of dysmenorrhea-related symptoms. It can also be considered that fever is more commonly associated with FMF patients due to elevated IL-1 levels.

It is also noteworthy that the effect of dysmenorrhea on daily life and school absenteeism was similar between FMF patients and controls. This finding is consistent with other studies, which have highlighted that the severity of menstrual pain does not always correlate with disruptions in daily activities, suggesting that individuals may develop coping strategies to manage the condition [38, 39].

Research highlights a complex relationship between BMI and dysmenorrhea in adolescents, influenced by physiological, hormonal, and lifestyle factors. A 2023 study by Jusuf et al. identified a significant association between underweight BMI and primary dysmenorrhea, with underweight adolescents showing higher odds of menstrual pain compared to those with normal BMI [40]. In 2023 Donayeva et al. noted that both underweight BMI and obesity were closely related to dysmenorrhea [41]. Similarly, El-Kosery et al. added that both underweight and obese individuals were more prone to dysmenorrhea compared to those with normal BMI, affecting school attendance and social activities [42]. Contrasting findings were presented by Vilšinskaitė et al., who reported no direct correlation between BMI and dysmenorrhea severity [43]. In this study, we compared dysmenorrhea features between BMI-matched groups, excluding underweight and obese individuals to eliminate the confounding effect of BMI. By matching patients on BMI, we aimed to minimize these potential confounding effects and isolate the variables of primary interest, providing a more focused analysis of dysmenorrhea’s underlying mechanisms. We acknowledge that matching by BMI limits the generalizability of our findings to populations with a broader BMI range. This was a deliberate trade-off, as our primary goal was to reduce heterogeneity in the sample and ensure that the observed associations were not disproportionately influenced by weight-related factors.

### Limitations

This study has some limitations that must be acknowledged. The reliance on self-reported data for menstrual symptoms introduces potential recall bias. However, given that the menstrual cycle is a recurring monthly phenomenon, it was assumed that participants would not face significant difficulty recalling symptoms associated with dysmenorrhea. Additionally, the cross-sectional design precludes causal inferences about the relationship between FMF and menstrual symptoms.

### Conclusion

In conclusion, this study highlights the intricate relationship between FMF and menstrual health, providing insights into dysmenorrhea characteristics, genetic influences, and quality-of-life impacts in adolescent girls. When dealing with patients’ chronic inflammatory conditions and long-term morbidities, it is also important to question components of normal adolescent development, such as the menstrual cycle. By integrating our findings with the existing literature, we highlight the need for a holistic, multidisciplinary approach that addresses both inflammatory and reproductive health needs in the management of FMF in adolescent patients, which will be important to optimize the care and improve the quality of life of this patient population.

## Data Availability

The data underlying this article will be shared on reasonable request to the corresponding author.

## Abbreviations

FMF: Familial Mediterranean fever
IL-1: Interleukin-1
IQR: Interquartile range
*MEFV*: Mediterranean fever gene
ELE: Erysipelas-like erythema ()
PRINTO: Pediatric Rheumatology International Trials Organization
BMI: Body mass index
